# Efficacy, safety and lack of immunogenicity of insulin aspart compared with regular human insulin for women with gestational diabetes mellitus

**DOI:** 10.1111/j.1464-5491.2007.02247.x

**Published:** 2007-10

**Authors:** D. J. Pettitt, P. Ospina, C. Howard, H. Zisser, L. Jovanovic

**Affiliations:** Sansum Diabetes Research Institute Santa Barbara, CA; * Novo Nordisk Inc. Princeton, NJ, USA

**Keywords:** diabetes, insulin, insulin analogues, pregnancy

## Abstract

**Aim:**

The efficacy and safety of insulin aspart (IAsp), a rapid-acting human insulin analogue, were compared with regular human insulin (HI) as the bolus component of basal-bolus therapy for subjects with gestational diabetes mellitus (GDM).

**Methods:**

In a randomized, parallel-group, open-labelled trial, 27 women with GDM (age 30.7 ± 6.3 years, HbA_1c_ < 7%) were randomized to receive IAsp (5 min before meal) or HI (30 min before meal). The trial period extended from diagnosis of GDM (18–28 weeks) to 6 weeks postpartum.

**Results:**

Both treatment groups maintained good overall glycaemic control during the study (beginning and end of study HbA_1c_≤ 6%). During the meal test, mean glucose at week 6 (IAsp 4.2 ± 0.57 mmol/l, HI 4.8 ± 0.86 mmol/l) was slightly lower than at week 0 (IAsp 4.9 ± 0.59 mmol/l, HI 5.1 ± 0.36 mmol/l). However, change from baseline values for average glucose (IAsp –1.09 ± 0.54 mmol/l, HI –0.54 ± 0.74 mmol/l; *P* = 0.003) and C-peptide (IAsp –0.50 ± 0.67 nmol/l, HI –0.30 ± 0.70 nmol/l; *P* = 0.027) were significantly lower after IAsp treatment than HI treatment. No major hypoglycaemic events were reported during the study. Cross-reacting insulin antibody binding increased slightly from baseline in both treatments groups (end of study: IAsp 2.1 ± 5.4%, HI 6.4 ± 13.9%), whereas antibodies specific to IAsp or HI remained relatively low (< 1% binding).

**Conclusion:**

IAsp was more effective than HI in decreasing postprandial glucose concentrations. Duration of IAsp injection 5 min before a meal rather than 30 min prior to meals offers a more convenient therapy for subjects with GDM. Overall safety and effectiveness of IAsp were comparable to HI in pregnant women with GDM.

Diabet. Med. 24, 1129–1135 (2007)

## Introduction

Gestational diabetes mellitus (GDM) is defined as glucose intolerance that occurs during pregnancy [1–3]. GDM affects about 7% of all pregnancies, resulting in > 200 000 cases per year [2]. Depending on the population sample and diagnostic criteria, the prevalence may range from 1 to 14%[1,2]. Of all the pregnancies complicated by diabetes, GDM accounts for about 90%[1]. Pregnancy complicated by GDM increases the risk of both maternal and neonatal complications. Women with GDM are at high risk of developing overt Type 2 diabetes months or years postpartum [4,5]. The most common and significant neonatal complication associated with GDM is macrosomia [6], which occurs at rates as high as 40% of neonates in untreated GDM [7]. In addition, neonatal macrosomia is associated with the metabolic syndrome of hyperinsulinaemia and deposition of fat in the visceral cavity [8]. Other neonatal complications include hypoglycaemia, hyperbilirubinaemia, hypocalcaemia, and erythraemia [9].

The diagnosis of GDM can be made based on the 75-g, 2-h oral glucose tolerance test (OGTT) [10] or on 100-g, 3-h OGTT criteria developed by the National Diabetes Data Group as modified by Carpenter and Coustan [11]. Tight glycaemic control both pre- and postprandially is of critical importance and has been shown to have an impact on neonatal outcome [12,13]. The standard treatment approach for GDM is an intensified insulin regimen if diet therapy alone fails to restore adequate glycaemic control [fasting blood glucose (BG) < 5.0 mmol/l (90 mg/dl) and 1-h postprandial BG < 6.7 mmol/l (120 mg/dl) determined from capillary blood][14]. Adequate insulin therapy should not only be effective in achieving glycaemic control, but also utilize formulations that have a low immunogenic potential [15]. As shown by Menon *et al.*, insulin–antibody complexes may cross the placenta and be associated with the development of macrosomia in the infant [15]. Since the high probability of development of Type 2 diabetes in females with a history of GDM may be explained on the basis of pregnancy-induced acceleration of pancreatic β-cell secretory exhaustion, treatment that would produce a β-cell-sparing effect on endogenous insulin secretion could prove beneficial.

Jovanovic *et al*. have documented that women with GDM who received a rapid-acting insulin analogue (insulin lispro) in a 6-week study had significantly lower postprandial increases in plasma C-peptide (and hence in endogenous insulin secretion) than those who injected regular human insulin [16]. In addition, better overall glycaemic control (mean decrease from baseline HbA_1c_: insulin lispro 0.35%, vs. regular insulin 0.07%; *P* < 0.005) was achieved in insulin lispro-treated individuals than in women receiving regular human insulin as a component of a basal-bolus regimen. Based on these findings, a hypothesis was proposed that use of a rapid-acting insulin analogue, insulin aspart (IAsp), may improve postprandial glycaemia during pregnancy complicated by gestational diabetes. This hypothesis sets the conceptual framework for studying IAsp in GDM. The short-term efficacy of IAsp has been demonstrated in a study of 15 women with GDM during standardized meal tests, where insulin aspart was shown to be effective in decreasing postprandial glucose concentration [17]. The present study further assesses whether IAsp is a safe and effective alternative to regular human insulin for overall glycaemic control in subjects with GDM. Thus, the primary end-points were adequate control of plasma glucose and lack of significant immunogenicity.

## Patients and methods

The study was performed with the approval of the Cottage Health Systems institutional review board at the study site. Written informed consent was obtained from all subjects before any trial-related activities were initiated. In this single-centre, randomized, parallel-group, open-label trial, 27 women (age 30.7 ± 6.3 years, HbA_1c_ < 7.0% at diagnosis, able and willing to perform self-measured BG readings seven times a day and inject insulin at least four times a day) with GDM, were randomized to receive either IAsp (NovoLog®; Novo Nordisk A/S, Bagsvaerd, Denmark) 5 min before meal or regular human insulin (HI, Novolin R; Novo Nordisk A/S) 30 min before meal. Insulin was administered using the NovoPen® 3 injection device. Subjects also received insulin NPH (Novolin N; Novo Nordisk A/S) as the basal insulin. The trial period extended from the diagnosis of insulin requiring GDM (18–28th week of pregnancy) to 6 weeks postpartum.

The initial treatment regimen consisted of two daily doses (morning and bedtime) of NPH and three daily doses of either IAsp or HI according to the scale published by Jovanovic *et al*. [14]. Insulin administration was initiated at the time GDM was diagnosed and continued through the end of pregnancy. The initial total insulin dose was 0.8 U/kg per day if gestation < 26 weeks or 0.9 U/kg per day if gestation ≥ 26 weeks [17]. In addition to insulin therapy, subjects were instructed to follow a recommended dietary regimen of 30 kcal/kg if their current body weight was within 80–120% of ideal body weight, 24 kcal/kg if their weight was within 121–150% of their ideal weight, or 12 kcal/kg if their weight was > 150% of their ideal body weight.

Before the initiation of insulin therapy, subjects had standardized meal tests performed on three consecutive days (without insulin on day 1 and with IAsp and HI in random sequence on days 2 and 3) at the start of the trial. A second set of meal tests was performed on two consecutive days (with IAsp and HI in random sequence) after 6 weeks of basal-bolus treatment. All meal tests were conducted in the morning after an overnight fast. The mealtime tests were conducted in a crossover manner and were not limited to the specific mealtime insulin that subjects had been receiving during the study. A mixed meal test was chosen because it is more physiological than a glucose load, but is still standardized and reproducible. Determining the effect of prandial insulins on postprandial glucose required a standardized meal. Women were tested during different stages of gestation and the crossover design allowed each woman to serve as her own control. During the paired meal tests, both at baseline and at 6 weeks, the same dose of either HI or IAsp was given on both days and was two-ninths of the total daily insulin requirement [17]. The mixed meal test consisted of 40% carbohydrate, 20% protein and 40% fat and was calculated to provide 20% of the woman's daily caloric requirement [17]. Blood samples were drawn for plasma glucose, serum insulin and C-peptide determinations at the following time points: –30, 0, 15, 30, 60, 90, 120, 180 and 240 min after the meal, and for proinsulin at 0 min only. No other food was consumed during this time, but symptomatic hypoglycaemia was treated with oral glucose tablets. Since each woman had meal tests using IAsp and regular HI, the values from the two tests were compared using paired *t*-tests. Overall glycaemic control was assessed by HbA_1c_ after 6 weeks of treatment, during the 36–38th weeks of pregnancy and at 6 weeks postpartum. HbA_1c_ at screening was assessed at the local trial site and all the subsequent HbA_1c_values were assessed at the central laboratory (MRL International, Highland Heights, KY, USA).

Safety assessments were based on adverse events (AEs), hypoglycaemic episodes, insulin-specific antibodies and cross-reactive insulin antibodies. Subjects recorded meter-measured BG values and symptoms of hypoglycaemia associated with BG meter readings. A minor hypoglycaemic episode was defined as an episode with symptoms consistent with hypoglycaemia (i.e. palpitations, tiredness, sweating, strong hunger, dizziness, tremour, etc.) with a confirmed BG meter reading < 2.8 mmol/l (50 mg/dl) and which was managed by the subjects themselves. Serum samples for determination of IAsp and HI concentrations, as well as insulin antibody binding, were collected prior to initiation of insulin, after 6 weeks of insulin therapy, during the 36–38th weeks of pregnancy and at 6 weeks postpartum. Samples were obtained from fasting individuals to minimize interference from administered HI and IAsp. Antibody binding specific to IAsp or HI was determined by radioimmunoassay and was expressed as the percent bound radioactivity used to assay the sample in a subtraction assay [18–20]. The value of cross-reacting antibody binding was calculated as the total HI binding that was inhibited by IAsp.

At birth, cord blood samples were collected for analysis of insulin concentration (IAsp and HI) and insulin antibody binding.

### Statistical methods

Statistical analyses were based on the intent-to-treat population. Missing values of HbA_1c_ were imputed using the last observation carried forward. The parameters in the meal test (which followed a cross-over design) were analysed using paired *t*-tests. A 5% level of significance was used throughout the study. All analyses were carried out using the two-sided tests. Results are presented as mean ±sd. The glucose area under the curve (AUC), measuring the total glycaemic exposure, was calculated using the trapezoidal rule [21].

## Results

### Demographic characteristics and subject disposition

Subject demographics and baseline characteristics are shown in Table 1. Most subjects were Latino and the distributions of ethnicity were similar in the two treatment groups. The group randomized to regular insulin had a slightly higher body mass index but the difference was not statistically significant. Twenty-seven subjects were randomly assigned to receive either IAsp (*N* = 14) or HI (*N* = 13). Thirteen (93%) subjects in the IAsp group and nine (69%) in the HI group completed the study. Four subjects discontinued the study since they delivered early and one subject discontinued due to the inability during the meal test to provide adequate blood samples because of excessive clotting.

**Table 1:** Demographics and baseline characteristics

	Insulin aspart *N* = 14	Regular insulin *N* = 13
Age (years)*	31.6 ± 5.9	29.7 ± 6.9
Race, *n* (%):		
Black	0	1 (8)
White	4 (29)	1 (8)
Hispanic	9 (64)	11 (85)
Native American	1 (7)	0
Parity	2.2	2.7
Height (cm)*	157.6 ± 6.9	155.6 ± 5.5
Weight (kg)*	72.9 ± 13.1	80.8 ± 16.1
Body mass index (kg/m^2^)*	29.3 ± 4.7	33.2 ± 5.7
HbA_1c_(%)	5.1 ± 0.4	5.3 ± 0.3

* Values are mean ±sd.

Differences between treatment groups were not statistically significant for all parameters.

### HbA_1c_

Both treatment groups maintained good overall glycaemic control during the study (beginning and end of the study HbA_1c_values ≤ 6%). Mean HbA_1c_values for the IAsp and HI groups were 5.2% during the 36–38th week of pregnancy and 5.4% at 6 weeks postpartum.

### Glucose, C-peptide, insulin and proinsulin values during the meal test

**FIGURE 1:**
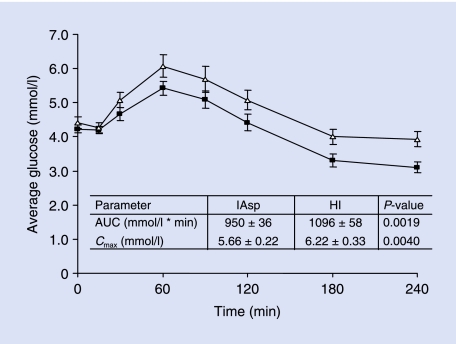
Average glucose profile during the meal tests. Average glucose (mmol/l) concentrations during the 4-h meal test with insulin aspart (IAsp) or regular insulin (HI). ▵, HI; ▪, IAsp.

The glucose profiles during the meal tests at week 6 are shown in Fig. 1. Thirty minutes after the meal, the women had a lower mean glucose concentration with IAsp than with HI (4.7 ± 0.19 mmol/l vs. 5.1 ± 0.23 mmol/l, respectively; *P* = 0.0278). The mean peak glucose concentration occurred at the 60-min time point for both treatment groups, but was significantly lower with IAsp than with HI (5.4 ± 0.21 mmol/l vs. 6.2 ± 0.33 mmol/l, respectively; *P* < 0.0097). The average glucose AUC and *C*_max_ values were significantly lower for IAsp than for HI (Fig. 1).

The average values for glucose, C-peptide, insulin and fasting proinsulin during the meal tests at baseline, week 0, and week 6 are summarized in Table 2. IAsp was effective in reducing the postprandial glucose concentration from baseline. The average glucose values during the meal test at week 6 were slightly lower than at week 0 for both treatment groups. However, treatment with IAsp during the meal test at week 6 led to lower glycaemic exposure than treatment with HI, as demonstrated by the significantly lower change from baseline glucose values (Table 2). The response to the follow-up meal tests was similar in the two groups, but both had lower peak glucose and lower AUC with IAsp. Both treatment groups had a slight increase in the average insulin at week 6 (reflecting the sum of endogenous insulin response plus exogenously administered mealtime insulin). However, the mean change from baseline insulin values at week 6 were similar for both treatment groups (Table 2). The contribution of endogenous insulin to the overall insulin profile was ascertained by measurement of C-peptide values, which were slightly lower for both IAsp and HI treatments at week 6 than at week 0. However, IAsp treatment resulted in a significantly lower C-peptide value at week 6 than did HI treatment (Table 2). The proinsulin concentrations for both treatments (reflecting overall endogenous insulin production) were lower at week 6 than at week 0 because of the lower demand for endogenous insulin after 6 weeks of treatment. However, the change from baseline values were not significantly different between treatments.

**Table 2:** Time-adjusted* average glucose, C-peptide, insulin and proinsulin values during the meal tests

		Observed data*		Change from baseline	
			
Week	Treatment‡	*N*	Mean ±sd	Mean ±sd	*P*-value§
Glucose (mmol/l)					
Baseline	No exogenous insulin	20	5.3 ± 0.43		
Week 0	Insulin aspart	19	4.9 ± 0.59	–0.41 ± 0.47	0.319
	Regular insulin	19	5.1 ± 0.36	–0.28 ± 0.34	
Week 6	Insulin aspart	15	4.2 ± 0.57	–1.09 ± 0.55	0.003
	Regular insulin	15	4.8 ± 0.86	–0.54 ± 0.74	
Insulin (pmol/l)					
Baseline	No exogenous insulin	20	210 ± 102		
Week 0	Insulin aspart	19	288 ± 120	78 ± 54	0.393
	Regular insulin	19	276 ± 138	60 ± 66	
Week 6	Insulin aspart	15	318 ± 96	114 ± 84	0.611
	Regular insulin	15	306 ± 120	108 ± 96	
C-peptide (nmol/l)					
Baseline	No exogenous insulin	20	1.50 ± 0.60		
Week 0	Insulin aspart	20	1.26 ± 0.46	–0.23 ± 0.23	0.076
	Regular insulin	19	1.36 ± 0.46	–0.17 ± 0.27	
Week 6	Insulin aspart	15	0.93 ± 0.46	–0.50 ± 0.67	0.027
	Regular insulin	15	1.13 ± 0.50	–0.30 ± 0.70	
Fasting proinsulin (pmol/l)†					
Baseline	No exogenous insulin	20	10.7 ± 20.6		
Week 0	Insulin aspart	20	9.7 ± 17.6	–1.1 ± 3.4	0.154
	Regular insulin	17	11.1 ± 21.8	–0.1 ± 1.6	
Week 6	Insulin aspart	14	3.8 ± 3.6	–3.0 ± 5.9	0.156
	Regular insulin	15	5.3 ± 5.2	–1.1 ± 5.9	

* The average values (determined at the time points: 30 and 0 min before the meal and at times 15, 30, 60, 90, 120, 180 and 240 min after the meal) were adjusted for possible differences in the duration of measurements and therefore represent the average concentration of the parameter over the time course of measurements.

† Proinsulin was measured only at time = 0 min before each meal test.

‡ Treatment received for meal test.

§ *P*-value for comparison between treatments was obtained from anova, with treatment included as fixed effect and baseline average values as covariate.

### Safety profile

Fourteen subjects reported a total of 27 AEs: eight (57%) subjects with 16 AEs in the IAsp group and six (46%) subjects with 11 AEs in the HI group. All reported AEs were not uncommon for the study population. In both treatment groups, the most frequently reported AE was upper respiratory tract infection [IAsp two (14%) subjects with two (12%) episodes; HI three (23%) subjects with three (27%) episodes]. The investigators considered fatigue (one subject) and somnolence (one subject) in the IAsp treatment group, and injection site reaction (IAsp, one subject; HI, two subjects) to be the only AEs possibly/probably related to the study drug.

### Hypoglycaemic episodes

Symptomatic hypoglycaemic episodes were reported by 19 subjects: 10 (71%) in the IAsp group (53 events) and nine (69%) in the HI group (23 events). Minor hypoglycaemic episodes (BG < 50 mg/dl) were reported by 16 subjects: 11 (79%) in the IAsp group (52 episodes) and five (39%) in the HI group (nine episodes). Half of the total minor hypoglycaemic episodes reported in the IAsp group were reported by two subjects prone to hypoglycaemia (subjects with 15 and 11 episodes). Most of the hypoglycaemic episodes occurred during the daytime hours (06.00–22.00 h) by 11 subjects (46 episodes) in the IAsp group and by five subjects (seven episodes) in the HI group. Four IAsp-treated subjects had six episodes of nocturnal hypoglycaemia (22.00–06.00 h) and one HI-treated subject had two episodes. No hypoglycaemic episodes required the assistance of another person.

### Insulin-specific antibodies and cross-reacting insulin antibodies

Cross-reacting antibody binding, human insulin-specific antibody binding and IAsp-specific antibody binding were determined before initiation of insulin therapy (visit 2), at 6 weeks of insulin therapy (visit 5), at 36–38th weeks of gestation (visit 7) and at 6 weeks postpartum (visit 9). Antibody binding specific to IAsp and HI remained relatively low for most of the subjects in both treatment groups throughout the study. At baseline, the mean (± sd) cross-reacting antibody binding was 0.2 ± 0.3% for both IAsp and HI groups. Mean cross-reacting antibody binding increased slightly to 1.4 ± 3.0% and 2.3 ± 5.4% for the IAsp group and 1.5 ± 2.3% and 6.5 ± 13.7% for the HI group, at visits 7 and 9, respectively. The increase in the mean value of the cross-reacting antibody binding for the IAsp group was largely attributable to one subject who had a binding value of 10.1% at visit 7 and a value of 19.2% at visit 9. One subject in the HI group had a similar increase in cross-reacting antibody binding of 47.0% at visit 9. However, these subjects did not require additional insulin to maintain normal overall glycaemic control (HbA_1c_at the end of the study < 5.3%).

### Examination of cord blood serum for the presence of insulin

Cord blood serum samples, collected immediately after delivery, were analysed for the presence of IAsp and HI. The lower limit of detection for HI was 11.0 pmol/l and for IAsp 5.3 pmol/l. During delivery, the IAsp concentration in the cord blood samples for three subjects in the IAsp treatment group (but not receiving intravenous IAsp during labour) were below the lower limit of detection. However, the IAsp concentration in the cord blood for one subject (randomized to the IAsp treatment group and receiving intravenous IAsp during labour) was 33 pmol/l. Four subjects in the HI treatment group showed an elevated level of HI in the cord blood serum sample that was particularly high in one subject (211 pmol/l) receiving intravenous HI treatment during labour.

In the cord blood serum samples, antibody binding specific to IAsp and to HI was similarly low for both treatment groups (< 0.5% binding of the specific antibodies). Although the IAsp concentration in the cord blood serum for one subject was 33 pmol/l, the antibody binding for the cord blood sample of this subject was low. Similarly, antibody binding in the cord blood serum sample that had a measurable HI concentration (211 pmol/l) also had low insulin-specific and cross-reacting antibody binding.

### Pregnancy outcome—neonatal assessment

The pregnancy outcomes (determined by the neonatal assessment: weight, length and physical examination findings) were similar in both treatment groups. The mean weights (IAsp 3.1 ± 0.5 kg; HI 3.0 ± 0.5 kg) and lengths (IAsp 49 ± 2.3 cm; HI 48 ± 2.4 cm) of the infants were similar between treatment groups. In this study, the maximum birth weight of an infant was 3.8 kg in the HI group and 4.1 kg in the IAsp group. No case of macrosomia was reported. Two infants in the HI group had abnormal physical examination findings (one infant diagnosed with Down's syndrome and one infant with petechiae on neck and upper chest from a tight umbilical nuchal cord). Another subject in the IAsp group had a fetus that died *in utero* during week 40 (fetal death due to umbilical cord strangulation). These events were deemed by the investigators as having an unlikely relationship to the study drug.

## Discussion

In this study, the greater reduction in the change from baseline average glucose values has demonstrated that subjects had better postprandial glycaemic control when they were treated with IAsp than with regular insulin. This finding is similar to a previous study using insulin lispro [16]. Studies in healthy volunteers have demonstrated that subcutaneously injected IAsp reaches peak plasma concentrations that are higher and occur earlier than those of regular HI, leading to a faster onset of blood glucose-lowering action [22–24]. Lindholm *et al*. [22] have shown that in healthy volunteers, the time to maximum serum glucose concentration after subcutaneous injection occurs > 1 h earlier for IAsp than for an equal dose of regular HI. Maternal postprandial glucose (1 h after beginning the meal) correlates positively with neonatal birth weight in pregnancies complicated by diabetes [25–27]. Adjustment of insulin therapy in gestational diabetes to normalize postprandial glucose levels leads to a decreased rate of macrosomia and lower rates of caesarean sections [27]. The neonatal birth weights were similar in the two groups and no case of macrosomia was reported.

Although more hypoglycaemic episodes were reported in the IAsp than in the HI treatment group, no major hypoglycaemic events (requiring the assistance of another individual) were reported in the study. Most of the hypoglycaemic episodes occurred during daytime hours, which allowed subjects to treat these episodes easily.

The mean change from baseline average plasma insulin values at week 6 was similar for the two treatment groups. The insulin assay used in this study measured total insulin (i.e. both exogenous and endogenous insulin). Both treatment groups had a slight increase in the insulin concentration between week 0 and week 6. This increase was not due to the endogenous insulin, as C-peptide levels were lower at week 6. The increase in insulin may be due to the increase in the total daily insulin dose resulting from the insulin dose titration.

The contribution of the endogenous insulin, measured by the C-peptide response, showed that the demand for endogenous insulin was lower after IAsp injection than after HI injection, despite the fact that the same dose of insulin was used. Such findings indicate that following sustained basal-bolus insulin therapy, less demand was placed upon the β-cells after IAsp injection than after HI injection. This observation probably reflects the fact that the pharmacokinetics of IAsp provides mealtime coverage of glycaemic needs by achieving higher peak insulin concentrations in less time and with a shorter duration of action than HI, thereby reducing the demand for endogenous insulin secretion during the meal test [28]. In this study, all subjects remained in good overall glycaemic control (HbA_1c_ at end of study was ≤ 6.0%).

Specific antibody binding to IAsp and regular HI remained relatively low (< 1.5% binding of specific antibody) for both treatment groups throughout the study. Increases in cross-reacting antibody binding have previously been observed in patients treated with IAsp and HI in a 12-month controlled trial in subjects with Type 1 diabetes [18]. However, the increase in the cross-reacting antibody binding at 3 months was transient and returned to near normal by 12 months. Although the reason for the rise in specific antibodies is unknown, the increase in antibodies was not accompanied by a deterioration of glycaemic control and did not require an increase in insulin dose for these subjects to maintain glycaemic control.

Earlier studies have shown that insulin–antibody complexes may cross the placenta and be associated with the development of macrosomia in the infant [15]. No data were previously available to show whether IAsp crossed the placenta during fetal development. In this study, a cord blood sample taken after delivery from one subject was found to contain IAsp (33 pmol/l). This was the only subject to receive IAsp during delivery. The presence of IAsp in the cord blood sample of this subject may have occurred as a result of the disruption in the uterine–placental barrier during delivery. Since the sample was collected during delivery, it is not possible to determine whether IAsp crosses the placenta during development of the fetus. Another insulin analogue, insulin lispro, did not cross the human placenta in an *in vitro* perfusion study [29]. Infusion of insulin during labour may be associated with increased insulin concentrations in the cord blood. Since there were only two subjects in this study who had insulin infusion during delivery, further study is necessary to determine the safety of such insulin infusion during labour. Overall, IAsp appeared to be of low immunogenicity in women with GDM.

In conclusion, this study has demonstrated that the overall safety and effectiveness of IAsp was comparable to regular HI in pregnant women with GDM. IAsp was more effective than regular HI in providing postprandial glycaemic control in women with GDM.

## Abbreviations

AE: adverse event
AUC: area under the curve
BG: blood glucose
GDM: gestational diabetes mellitus
HI: human insulin
IAsp: insulin aspart
OGTT: oral glucose tolerance test
